# Advances in Brain Metastases Diagnosis: Non-coding RNAs As Potential Biomarkers

**DOI:** 10.7759/cureus.36337

**Published:** 2023-03-18

**Authors:** Akram M Eraky

**Affiliations:** 1 Neurosurgery, Medical College of Wisconsin, Milwaukee, USA

**Keywords:** serum biomarkers, csf biomarkers, long non-coding rna, mirna, isolated brain metastasis, brain metastases with nsclc, solitary brain tumor metastases (sbms), non-coding rna, microrna, lncrna

## Abstract

Brain metastasis is considered the most common brain tumor. They arise from different primary cancers. The most common primary tumors giving brain metastases include breast, colorectal, lung, melanoma, and renal cancer. Depending only on history, physical examination, and conventional imaging modalities makes brain tumors diagnosis difficult. Rapid and non-invasive promising modalities could diagnose and differentiate between different brain metastases without exposing the patients to unnecessary brain surgeries for biopsies. One of these promising modalities is non-coding RNAs (ncRNAs). NcRNAs can determine brain metastases' prognosis, chemoresistance, and radioresistance. It also helps us to understand the pathophysiology of brain metastases development.

Additionally, ncRNAs may work as potential therapeutic targets for brain metastases treatment and prevention. Herein, we present deregulated ncRNAs in different brain metastases, including microRNAs and long non-coding RNAs (lncRNAs), such as gastric adenocarcinoma, colorectal, breast, melanoma, lung, and prostate cancer. Additionally, we focus on serum and cerebrospinal fluid (CSF) expression of these ncRNAs in patients with brain metastases compared to patients with primary tumors. Moreover, we discuss the role of ncRNAs in modulating the immune response in the brain microenvironment. More clinical studies are encouraged to assess the specificity and sensitivity of these ncRNAs.

## Introduction and background

Metastases are considered the most common brain tumors [1]. Brain metastases arise from primary cancers, such as breast, colorectal, lung, melanoma, and renal [1]. Of interest, according to the National Comprehensive Cancer Network (NCCN), screening for brain metastases by MRI is recommended only in the case of diagnosis of the following cancers: non-small cell lung cancer (NSCLC), small cell lung cancer, melanoma, testicular cancer, left cardiac sarcoma, angiosarcoma, and alveolar soft parts sarcoma [2].

Depending only on history, physical examination, and conventional imaging modalities makes brain tumors diagnosis difficult. Rapid and non-invasive promising modalities could diagnose and differentiate between different brain metastases without exposing the patients to unnecessary brain surgeries for biopsies. One of these promising modalities is non-coding RNAs (ncRNAs) [3,4]. NcRNAs have potential roles in determining brain metastases prognosis, chemoresistance, and radioresistance. It also helps us to understand the pathophysiology of brain metastases development. Additionally, ncRNAs may work as potential therapeutic targets for brain metastases treatment and prevention.

Only a small portion of the human genome is transcribed into protein-coding RNAs that can be translated into proteins. In contrast, most of our genome is considered non-protein-coding genes that are not expressed into proteins. Most non-protein-coding genes can be transcribed into ncRNAs, essential in regulating pre-and post-transcriptional steps in protein synthesis [5-8]. ncRNAs with more than 200 nucleotides are called long ncRNAs (lncRNAs), while ncRNAs with less than 200 nucleotides are called small ncRNAs [8]. MicroRNAs are considered small RNAs regulating messenger RNA (mRNA) translation by binding to mRNAs, inhibiting protein production [9,10].

LncRNAs regulate gene expression by different mechanisms [6,11-15]. First, lncRNAs can bind to transcription factors and recruit them to the promoter locus to induce gene transcription. The second mechanism by which lncRNAs can regulate gene expression is that lncRNAs can work as decoy molecules by binding to proteins regulating gene expression, such as transcription factors, subsequently suppressing gene expression [6,11-15]. Thirdly, lncRNAs induce chromatin modification by recruiting epigenetic modifications-inducing enzymes, such as histone methylase, deacetylase, and acetylase, resulting in the upregulation or downregulation of the target gene. Fourthly, lncRNAs can act as scaffolding proteins by binding to other proteins, such as heterogeneous nuclear RNA (hnRNA), to make RNA-protein complexes (RNPs) that can activate gene expression by binding to gene promoters or repress gene expression by binding to gene repressors [6,11-15].

**Figure 1:**
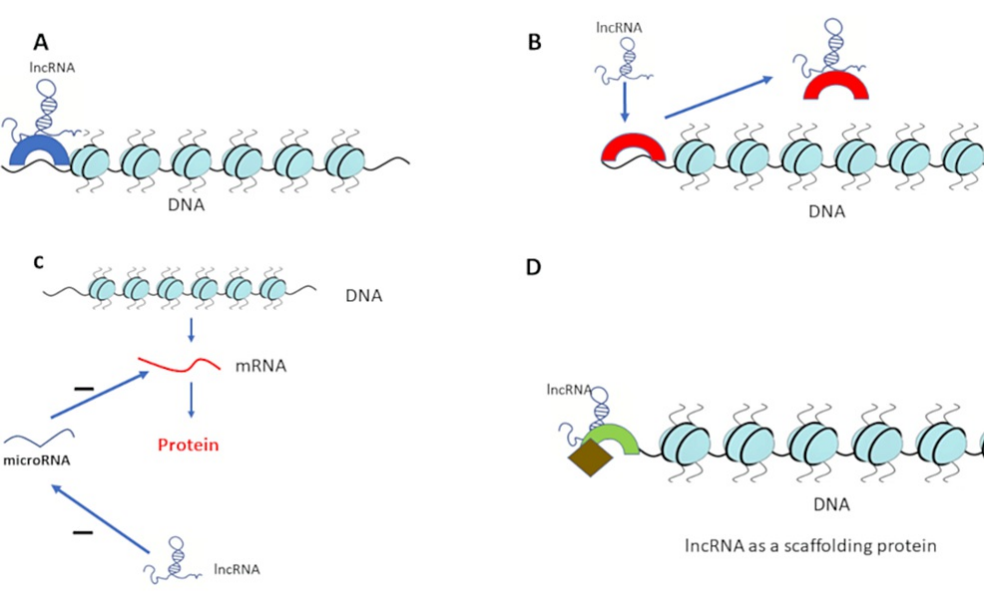
Regulation of gene expression by lncRNAs (A) Long non-coding RNAs (lncRNAs) can bind to transcription factors and recruit them to the promoter locus to induce gene transcription. (B) LncRNAs can work as decoy molecules by binding to proteins regulating gene expression, such as transcription factors, subsequently suppressing gene expression. (C) LncRNAs can act as competitive endogenous RNAs (ceRNAs) by sponging microRNAs, preventing microRNAs from binding to and inhibiting messenger RNA (mRNA). (D) LncRNAs can act as scaffolding proteins by binding to other proteins, such as heterogeneous nuclear RNA (hnRNA), to make RNA-protein complexes (RNPs) that can activate gene expression by binding to a locus for gene promoter or repress gene expression by binding to gene repressors [6,11-15]. Image credit: Akram M. Eraky.

Additionally, lncRNAs can act as miRNAs precursors by being processed into many microRNAs. Lastly, lncRNAs can act as competitive endogenous RNAs (ceRNAs) by sponging microRNAs, leading to preventing microRNAs from binding to and inhibiting messenger RNA (mRNA) [6,11-15]. Figure 1 illustrates these different mechanisms of lncRNAs.

## Review

Role of microRNAs in brain metastases diagnosis

Searching for new non-invasive biomarkers that can help diagnose metastases and predict prognosis and may work as therapeutic targets is a trend. In two previous reviews, we discussed the potential role of ncRNAs as possible biomarkers for the diagnosis, prognosis, radiosensitivity, and histopathologic grade of glioblastoma and meningioma [3,4]. Herein, we discuss the possibility of using ncRNAs as potential biomarkers for metastases diagnosis.

Recently, many ncRNAs have been deregulated in brain metastases compared to primary tumors. For instance, Nass et al. found that compared to brain metastases, microRNA-92b, microRNA-9, and microRNA-9* are upregulated in primary brain tumors [16]. This suggests using these microRNAs as possible biomarkers to differentiate between primary brain tumors and brain metastases [16]. Another study by Pangeni et al. found that many deregulated non-protein-coding genes are associated with brain metastases [17]. They found three of these genes are hypermethylated (MIR124-2, RP11-713P17.4, NUS1P3), while the other three genes, including CTD-2023M8.1, MIR3193, and MTND6P4, are found to be hypomethylated in brain metastases compared to primary brain tumors [17]. This suggests that epigenetic regulation of the genes coding for ncRNAs can affect their expression, leading to altered levels and inducing tumor cell invasion, proliferation, and migration.

Herein, we present deregulated ncRNAs in different brain metastases, including microRNAs and lncRNAs. Additionally, we focus on serum and cerebrospinal fluid (CSF) expression of these ncRNAs in patients with brain metastases compared to patients with primary tumors.

Role of MicroRNAs in Non-Small Cell Lung Cancer (NSCLC) Brain Metastases

Regarding NSCLC brain metastases, Tsakonas et al. studied microRNA expression in patients with NSCLC primary tumors with metastatic brain NSCLC [18,19]. They found that compared to primary NSCLC tumor samples, there are five microRNAs upregulated (microRNA-219a-5p, microRNA-9-5p, microRNA-124-3P, microRNA-219a-2-3p, and microRNA-129-2-3p) in brain metastases, while six microRNAs are found to be downregulated (microRNA-199a-5p, microRNA-199b-5p, microRNA-142-3p, microRNA-199b-5p, microRNA-199a-3p, and microRNA-150-5p) [18,19]. In adenocarcinoma metastases, Zhao et al. found that five microRNAs are upregulated ( microRNAs-9*, microRNA-1471, microRNA-718, microRNA-3656, microRNA-720) compared to primary lung adenocarcinoma, while three microRNAs were found to be downregulated (microRNAs-214, microRNA-145, microRNA-23a) [20].

Compared to NSCLC cell lines with low metastatic potential, Wang et al. found that those with high metastatic potential have significantly downregulated microRNA-590. They also found that inducing microRNA-590 expression inhibits tumor cell proliferation, migration, and invasion [21]. In another study, Donzelli et al. found reduced expression of microRNA-145-5p in vitro and in vivo stimulated cancer cell migration [22]. This suggests that microRNA-145-5p is important in malignant cell proliferation, invasion, and migration. Another study by Chen et al. found that microRNA-375 was significantly downregulated in patients with brain metastases compared to NSCLC patients without brain metastases [23]. They also suggested that the poor prognosis in patients with low microRNA-375 may be due to matrix metalloproteinase-9 (MMP-9) and vascular endothelial growth factor (VEGF) overexpression [23].

Liu et al. studied the relationship between hypoxia and microRNA-155 [24]. They found hypoxia induces microRNA-155 release by increasing hsp70 production from lung cancer cells [24]. Upregulated microRNA-155 inhibits occludin protein expression, increasing blood-brain-barrier (BBB) permeability and subsequently increasing lung cancer brain metastases [24]. Of interest, Singh et al. found that STAT3 knocking down can reduce lung cancer cell migration by its direct effect on microRNA-21 [25]. This suggests using STAT3 as a potential therapeutic target to prevent brain metastases.

Role of MicroRNAs in Gastric Adenocarcinoma (GA) Brain Metastases

Studying microRNAs expression in primary gastric adenocarcinoma (GA) and brain metastases tissue samples, Minn et al. found that compared to primary GA, there are 6 microRNAs significantly upregulated in brain metastases samples (microRNA-106b-5p, microRNA-1260a, microRNA-141-3p, microRNA-19a-3p, microRNA-200b-3p, microRNA-93-5p). In comparison, two microRNAs are found to be downregulated (microRNA-4430, microRNA-4689) [26].

Role of MicroRNAs in Breast Cancer Brain Metastases

Regarding brain metastatic breast cancer, Ulasov et al. found that microRNA-345 suppresses KISS1 expression; subsequently, it induces E-cadherin expression. They also found that metastatic breast cancer cells have higher expression of E-cadherin and reduced expression of KISS1 [27]. This highlights the potential role of microRNA-345 as a therapeutic target to decrease metastatic breast cancer cell migration, invasion, and proliferation [27]. Similar results are reported by Kaverina et al. [28]. Zhang et al. found that microRNA-1258 has an anti-oncogenic role in patients with breast cancer brain metastases [29]. They suggest this effect may happen through the microRNA-1258 inhibition effect on heparinase, a potent metastasis-inducing enzyme overexpressed in brain metastases [29]. This shows the potential role of microRNA-1258 as a therapeutic target for brain metastases treatment.

Harati et al. found that in brain metastatic breast cancer cells, microRNA-101-3p is downregulated. They also found that microRNA-101-3p inhibition induces metastatic cell migration, while microRNA-101-3p upregulation inhibits metastases formation by modulating COX-2 expression [30]. Another study by Ahmad et al. found that microRNA-10b overexpression was observed in patients with developed brain metastases compared to patients with breast cancer who did not develop brain metastases [31]. This suggests using microRNA-10b as a biomarker for breast cancer brain metastases and a prognostic factor to assess the effect of anticancer medications.

In another study by Gravgaard et al., they found that the expression of fifteen microRNAs significantly differs between the primary breast cancers versus the metastatic breast cancer cells [32]. Compared to the primary breast tumors, thirteen microRNAs were found to be upregulated in metastatic cells (microRNA-200a, microRNA-200b, microRNA-200c, microRNA-141, microRNA-9, microRNA-219-5p, microRNA-1274a, microRNA-Plus-E1136, microRNA-Plus-E1088, microRNA-525-pre, microRNA-Plus-G1248-5p, microRNA-Plus-G1307-pre, and miRNA-Plus-G1249-5p), while microRNA-202 and microRNA-Plus-E1133 were found to be downregulated [32]. This suggests the involvement of these microRNAs in metastatic cells' proliferation, migration, and invasion.

By inducing overexpression or knocking down microRNA-141 by lentiviral vectors, Debeb et al. found that downregulated microRNA-141 inhibited metastatic cell migration and invasion, while microRNA-141 overexpression was associated with severe metastatic activity [33]. In another study by Giannoudis et al., three microRNAs are downregulated in metastases cells (microRNA-132-3p, microRNA-199a-5p, microRNA-150-5p, microRNA-155-5p) compared to primary breast cancer cells, while microRNA-132-3p was found to be upregulated [34]. This suggests the potential role of these microRNAs as biomarkers for metastases diagnosis.

Ahmad et al. found that compared to primary breast tumors, microRNA is upregulated in breast cancer brain metastases [35]. They also found that microRNA-20b is overexpressed in breast cancer brain metastases compared to breast cancer bone metastases [35]. This suggests the significant role of microRNA-20b in brain metastases development. Another study by Harati et al. found that microRNA-202-3p knocking down in breast cancer cells induces cancer cell migration and invasion through the brain endothelium by MMP-1 upregulation and interepithelial junction disruption [36]. They found that microRNA-202-3p upregulation inhibits tumor cell migration [36].

Role of MicroRNAs in Colorectal Cancer Brain Metastases

Compared to primary colorectal cancers, Li et al. found that upregulation of microRNA-10b, microRNA-22, microRNA-133b, microRNA-145*, microRNA-145, microRNA-1, microRNA-146a, microRNA-576-5p, microRNA-126*, HS287, microRNA-28-5p, microRNA-143, microRNA-199b-5p, microRNA-199a-5p, microRNA-199a, microRNA-133a, and microRNA-125b and down-regulation of microRNA-31 and HS170 are observed in colorectal brain metastases [37]. This suggests that these microRNAs are essential in metastases development and indicates their potential use as possible biomarkers for metastases diagnosis.

Roskova et al. studied microRNAs expression in brain metastases arising from different origins, and they found that there is a significantly different expression of six microRNAs in five groups of brain metastases, including brain metastases with origin in melanomas, renal cell carcinomas, breast carcinomas, lung carcinomas, colorectal carcinomas [38]. These microRNAs include microRNA-141-3p, microRNA-141-5p, microRNA-146a-5p, microRNA-194-5p, microRNA-200b-3p, microRNA-365b-5p [38].

Role of microRNAs in the radiosensitivity of metastatic cells

In brain metastatic breast cancer, Ulasov et al. found that microRNA-345 suppresses KISS1 expression; subsequently, it induces E-cadherin expression. Of interest, they found that exposure of metastatic cells lacking KISS1 to radiation or temozolomide led to cell proliferation compared to control cells having normal levels of KISS1 [27]. They also found that E-cadherin levels are high in cells exposed to radiation or temozolomide [27]. This suggests a potential therapeutic target to treat radiation and temozolomide-resistant metastatic cells by increasing KISS1 expression through downregulating microRNA-345.

Role of microRNAs as serum biomarkers

Using an animal model, Sereno et al. found that microRNA-194-5p and microRNA-802-5p are downregulated, while microRNA-92a-1-5p, microRNA-205-5p, and microRNA-181a-1-3p are found to be downregulated before developing breast cancer brain metastases [39]. This suggests the role of these microRNAs in malignant cell migration and invasion and highlights its potential role as a possible biomarker for metastasis prediction. In another study, Figueira et al. found many deregulated microRNAs in animals' plasma samples, such as microRNA-802-5p, microRNA-194-5p, microRNA-92a-1-5p, microRNA-205-5p, and microRNA-181a-1-3p before developing brain metastases, suggesting that these microRNAs have a vital role in metastases development [40]. They also suggested some microRNAs may help tumor cell transmission through BBB [40].

In patients with brain metastatic NSCLC, Dong et al. found that microRNA-21 serum levels are higher than in patients with primary NSCLC without brain metastases [41]. Among those patients, without brain metastatic NSCLC, 26 brain metastatic NSCLC cases with high microRNA-21 were newly diagnosed [41]. They suggested that this significant positive correlation between microRNA-21 serum levels and developing brain metastases highlights the possibility of using microRNA-21 as a serum biomarker for brain metastases diagnosis and prediction in patients with primary NSCLC [41]. They found that the best microRNA-21 cutoff point is 0.205, with a sensitivity of 92.3% and specificity of 60.7% [41]. They found that microRNA-21 inhibition restrains tumor cell proliferation, invasion, migration, and angiogenesis [41]. This demonstrates that microRNA-21 may be a potential therapeutic target for brain metastases.

Curtaz et al. studied the serum levels of microRNA-576-3p and microRNA-130a-3p in patients with breast cancer brain metastases. They found increased serum levels of microRNA-576-3p and decreased levels of microRNA-130a-3p in those patients. They also found that lower microRNA-342-3p levels correlate with worse tumor stages [42]. Calculating serum levels of microRNA-424 in patients with NSCLC brain metastases, Deng et al. found that microRNA-424 expression is higher in patients with chemoresistance [43]. This highlights the potential role of microRNA-424 in developing chemoresistance.

Role of microRNAs as cerebrospinal fluid (CSF) biomarkers

Calculating the CSF levels of microRNA- let-7b, micrRNA-21-3p, and microRNA-10a, Kopkova et al. found that the diagnostic score threshold for brain metastases is -2.164 with a sensitivity of 75% and specificity of 71% [44]. In another study, Teplyuk et al. found that microRNA-10b and microRNA-21 are upregulated in the CSF samples of patients with GBM or brain metastases [45]. Of interest, microRNA-10b is considered the only undetectable microRNA in the normal brain [45]. They also found that CSF levels of microRNA-200a, microRNA-200b, microRNA-200c, and microRNA-141 are high only in patients with brain or leptomeningeal metastases [45]. Also, microRNA-200a and microRNA-200b CSF levels are higher in patients with breast cancer metastases than in lung cancer metastases. In contrast, microRNA-141 and microRNA-200c CSF levels are similar in both metastases [45]. This suggests using the ratio between the CSF levels of these microRNAs to identify the type of brain metastases.

Role of microRNAs in differentiating between glioblastoma multiforme (GBM) and solitary brain metastasis

In both patients with GBM and NSCLC patients, Jin et al. found high serum levels of microRNA-221 [46]. They also found that in GBM patients, both microRNA-608 and microRNA-504 serum levels are significantly downregulated; however, microRNA-504 serum level was the only reliable biomarker to differentiate GBM versus NSCLC with a sensitivity of 100% and specificity of 88.89% [46]. In another study by Baraniskin et al., they found that microRNA-15b and microRNA-21 CSF levels are significantly higher in patients with gliomas than in patients with brain metastases [47].

Role of lncRNAs in brain metastases diagnosis

Wu et al. found that lncRNA-MMP2-2 increases BBB permeability, subsequently inducing NSCLC metastases. They also found that lncRNA-MMP2-2 inhibition reduces brain metastases formation in vivo [48]. Zheng et al. found that lncRNA-NNT-AS1 inhibition attenuates tumor cell proliferation, invasion, and migration by increasing miRNA-494-3p expression, subsequently decreasing PRMT1 levels [49]. Regarding lung adenocarcinoma brain metastases, Jiang et al. found that lnc-REG3G-3-1 is upregulated in brain metastases compared to primary adenocarcinoma tissues [50]. They found that lnc-REG3G-3-1 regulates leptin and SLC2A5 proteins by sponging microRNA-215-3p [50]. This suggests the potential role of lnc-REG3G-3-1 in brain metastases diagnosis and its possible role as a therapeutic target.

Regarding brain metastases affecting males more than females, Shen et al. found that miRNA-494-3p has an anti-oncogenic role in prostate cancer by inhibiting cancer cell migration and invasion [51]. This highlights the possibility of using lncRNA-494-3p inhibition as a potential therapeutic target to prevent brain metastases. In another study by Schmidt et al., they found that lncRNA- SLNCR1binds to both androgen receptor (AR) and transcription factor Brn3a to form a complex that increases MMP9 expression that induces melanoma cell invasion and migration [52]. The role of androgen receptors may explain why males with melanoma have a worse prognosis and higher risk of developing aggressive brain metastases than females with melanoma.

Regarding breast cancer, Wang et al. found that lncRNA-BM induces STAT 3 phosphorylation by increasing JAK2 kinase activity, subsequently increasing ICAM1 and CCL2 expression, which helps leukocytes adhesion to endothelium, macrophages recruitment, and IL-6 production [53]. This may explain how breast cancer cells adhere and migrate to the brain parenchyma [53]. Of interest, they found that inhibition of JAK2, STATA3, or lncRNA-BM may prevent breast cancer brain metastases [53]. In another study by Xing et al., they found that lncRNA- X-inactive specific transcript (lncRNA-XIST) is downregulated in breast cancer brain metastases compared to breast cancer bone metastases [54]. They also showed that lncRNA-XIST knocking down stimulated breast cancer cells' migration, proliferation, epithelial-mesenchymal transition, and invasion through increasing microRNA-503 expression that enhances tumor growth, MSN-c-Met activation, and microglia reprogramming [54]. Of interest, they suggested that lncRNA-XIST has an anti-oncogenic effect and represents a potential therapeutic target for brain metastases treatment [54]. They also found that fludarabine, a BBB permeable chemotherapy with an unclear mechanism of action, is more effective in decreasing the proliferation and migration of breast cancer cells with low levels of lncRNA-XIST [54]. To clarify the interaction between metastatic cells and the immune reaction in the brain, Liu et al. found that lncRNA-BMOR is an oncogenic lncRNA that helps metastatic cells to avoid immune-mediated killing and disruption in the brain parenchyma by binding to and inhibiting interferon regulatory factor 3 (IRF3), subsequently attenuating the immune response against the metastatic cells [55].

## Conclusions

NcRNAs have an essential role in regulating tumor-suppressor and tumor-inducing genes. Many ncRNAs have been found to have a diagnostic role in identifying brain metastasis, differentiating between different brain metastases, and recognizing radioresistant and chemoresistant metastases. Assessing the sensitivity and specificity of these ncRNAs in patients' serum and CSF should be encouraged because of the potential ability of ncRNAs to act as rapid and non-invasive biomarkers for metastases diagnosis.
